# Discovering putative novel stem rust resistance loci in wheat genetic resources

**DOI:** 10.1002/tpg2.70259

**Published:** 2026-06-04

**Authors:** Jiaojiao Wang, Guoliang Li, Tim Kloppe, Kerstin Flath, Philipp Schulz, Mathias Wiegman, Thomas Miedaner, Yong Jiang, Jochen C. Reif

**Affiliations:** ^1^ Leibniz Institute of Plant Genetics and Crop Plant Research (IPK) Seeland Germany; ^2^ Institut für Pflanzenschutz in Ackerbau und Grünland Julius Kühn‐Institut (JKI) Kleinmachnow Germany; ^3^ RAGT2n Silstedt Germany; ^4^ State Plant Breeding Institute University of Hohenheim Stuttgart Germany

## Abstract

Stem rust (SR), caused by *Puccinia graminis* f. sp. *tritici* (Pgt), poses a threat to wheat production in Germany. This study evaluated 200 plant genetic resources (PGR) and 50 elite lines across four field environments. Multi‐environment genome‐wide association mapping based on PGR accessions revealed 1942 marker‐trait associations (MTAs), grouped into eight quantitative trait loci (QTLs), which explained 0.84%–28.19% of phenotypic variance. Three of these QTL, *QSr1B_1.47_67.79*, *QSr4A_716.31_716.32*, and *QSr7D_171.80_174.34*, harbor five haplotypes that have likely not been used in breeding. Five representative MTAs (Chr1B:25506861, Chr1B:17349639, Chr1B:67755088, Chr4A:716319007, and Chr7D:171807054) represent these five putative novel haplotypes, and together they conservatively explained 2.40%–7.03% of the phenotypic variance. Four genotypes carrying all favorable alleles of the representative MTAs were identified as potential donor lines for future breeding. Within eight QTL regions, 44 candidate genes were prioritized. These findings provide new insights into the genetic architecture of SR resistance and highlight promising targets for marker‐assisted prebreeding.

## Plain Language Summary

Stem rust (SR) is a serious disease that can cause major yield losses in wheat. Recent outbreaks in Europe have shown that the risk of SR is increasing, highlighting the need to find new sources of resistance for European wheat breeding. In this study, we used genome‐wide association studies to identify three genetic regions, quantitative trait loci, containing five haplotypes that have likely not yet been used in breeding. We also identified four donor lines that carry all five haplotypes, making them promising candidates for future breeding work. This study provides a data‐driven approach that can be applied to discover and use valuable genes from gene banks for improving complex traits in wheat and other crops.

**Abbreviations**
ASR: all‐stage resistance
BLUEs: best linear unbiased estimators
FAF: favorable allele frequency
G×E: genotype‐by‐environment
GWAS
: genome‐wide association study
IPK: Leibniz Institute of Plant Genetics and Crop Plant Research
LD: linkage disequilibrium
LMM: linear mixed model
MAF: minor allele frequency
MTA: marker‐trait association
PGR: plant genetic resources
Pgt: *Puccinia graminis* f. sp. *tritici*
PVE: phenotypic variance explained
QTL: quantitative trait locus
SNP×E: SNP‐by‐environment interaction
SNPs: single‐nucleotide polymorphisms
SR: stem rust
T3C: trait‐customized core collection
WGS: whole‐genome resequencing

## 1 INTRODUCTION

Stem rust (SR), caused by *Puccinia graminis* f. sp. *tritici* (Pgt), is one of the most devastating diseases of wheat (*Triticum aestivum* L.), capable of rapid development and severe yield losses worldwide (Kayim et al., 2022). Although SR had been absent in Germany for decades, it re‐emerged in five federal states in 2013 (Olivera Firpo et al., 2017), prompting renewed research interest (Flath et al., 2018). Breeding for resistant cultivars remains a key strategy for mitigating SR outbreaks. A recent study confirmed that major resistance genes *Sr24*, *Sr31*, and *Sr38*/*Yr17* are still effective in German wheat germplasm; however, no new major resistance loci have been identified in local varieties (Miedaner et al., 2024). Notably, highly virulent Pgt races such as TTKST and TKKTP were not accounted for in Miedaner et al. (2024), despite their known ability to overcome *Sr24* and *Sr38/Yr17*, highlighting a critical gap in current resistance deployment strategies (Flath et al., 2018; Jin et al., 2007; Olivera Firpo et al., 2017).

To date, >60 resistance genes for SR have been designated, and only a subset have been cloned (*Sr6*, *Sr9*, *Sr13*, *Sr21*, *Sr22, Sr26, Sr27, Sr33*, *Sr35*, *Sr43*, *Sr45, Sr50*, *Sr55*, *Sr57*, *Sr60*, *Sr61*, and *Sr62*) (McIntosh et al., 2020; Y. Wang et al., 2024; Yu et al., 2023; J. Zhang et al., 2023). The majority of these genes confer race‐specific all‐stage resistance (ASR) and exhibit limited durability (McDonald & Linde, 2002). Most SR genes possess only a few functional alleles; however, *Sr9* represents an exception, showing extensive allelic diversification (*Sr9a*, *Sr9b*, *Sr9d*, *Sr9e*, *Sr9f*, *Sr9g*, and *Sr9h*) (J. Zhang et al., 2023). In addition, >300 quantitative trait loci (QTL)—not all of which are unique—have been associated with SR resistance in wheat (Cheng et al., 2024; Miedaner et al., 2024; Pal et al., 2022; Tong et al., 2024).

Diversifying the genetic basis of SR resistance of the European elite wheat breeding pool by identifying novel resistance sources (Saunders et al., 2019) and accumulating multiple resistance genes (Mundt, 2018; Njau et al., 2013) is essential for mitigating the impact of rapidly evolving pathogens. Plant genetic resources (PGR) conserved at the German *Federal Ex situ Gene Bank*, operated by the *Leibniz Institute of Plant Genetics and Crop Plant Research* (*IPK*), represent a rich and underexplored reservoir for discovering new SR resistance alleles. Targeted screening for favorable alleles conferring resistance to yellow rust (*Puccinia striiformis* f. sp. *tritici*) and leaf rust (*Puccinia triticina*) has been successfully pursued in previous studies using PGR (Kale et al., 2022; Schulthess, Kale, Liu, et al., 2022). These efforts led to the development of a trait‐customized core collection (termed T3C population), which has proven valuable for yellow rust and leaf rust resistance studies (Schulthess, Kale, Liu, et al., 2022). However, the potential of the T3C population for identifying novel SR resistance alleles remains unexplored and represents a critical opportunity for advancing durable resistance breeding. In this study, we employed genome‐wide association mapping to dissect the genetic architecture of SR resistance in a diverse panel of 194 winter wheat genetic resources based on pre‐screening of the T3C population. Our objectives were threefold: (i) to identify genomic loci associated with SR resistance; (ii) to assess the potential of PGR for uncovering novel resistance sources not currently utilized in elite breeding programs; and (iii) to prioritize high‐confidence candidate genes within the genomic intervals of QTL.

## 2 MATERIALS AND METHODS

### 2.1 Plant material and field trial

The plant material used in this study is a subset of a T3C, which was introduced earlier (Schulthess, Kale, Liu, et al., 2022). Briefly, the T3C population comprised 200 elite varieties and 600 PGR accessions, which were selected from a large winter wheat collection maintained at the *German Federal Ex Situ Gene Bank* at IPK. Selection was based on molecular and phenotypic diversity for resistance to yellow rust, leaf rust, and powdery mildew (*Blumeria graminis*) in field trials from a previous study (Schulthess, Kale, Liu, et al., 2022). In this study, we focused on SR resistance, and we first pre‐screened the entire T3C by conducting unreplicated field evaluations with artificial inoculation at a single location over 2 years. Phenotypic data were available for 480 of the 600 PGR and 123 of the 200 elite lines (data not shown). A two‐tailed selection strategy, with emphasis on the resistance tail, was applied, resulting in the selection of 200 PGR and 50 elite lines for subsequent analyses.

The main experiment in this study was conducted with the 200 PGR and 50 elite varieties, which were evaluated in the field trial for resistance to SR in four environments (Table S1): Dahlem (52°27′29.5″ N, 13°17′56.3″ E) in 2023, Silstedt (51°51′16.0″ N, 10°51′37.0″ E) in 2023 and 2024, and Hohenheim (48°42′42.0″ N, 9°11′13.0″ E) in 2024. In each environment, the material was randomized in a separate trial using a resolvable incomplete block design with two complete replicates. Field trials were artificially inoculated with a mix of Pgt (Table S2). Isolates were mixed in equal proportions prior to inoculation. Inoculation was performed from the end of heading to mid‐flowering by applying a spore suspension with a microsprayer directly onto the plants from above across the entire field. Phenotyping was initiated when susceptible varieties exhibited clear symptoms, and it was conducted by the following visual scoring approach: The stem segment between the second leaf from the top and the adjacent internode was visually scored on a plot basis as the percentage of stem area covered by rust (0%–100%) and subsequently converted into a 1–9 scale (1 = 0–0.5%, 9 ≥ 70%). Scoring was assessed twice during the adult plant stage until full ripening, and the maximum value from the two assessments was used as the final measure of SR severity. Whole‐genome sequencing of Pgt samples (16 × genome coverage) collected from all environments was performed. Samples that did not meet quality criteria for any given environment were excluded from the analysis (Table S3). This approach provided preliminary insights into the potential impact of local pedoclimatic conditions on the genetic composition of the Pgt inoculum.

### 2.2 Phenotypic data analyses

For each environment, phenotypic analysis was conducted based on the following linear mixed model (LMM):

(1) $$y_{ikl}=\mu +g_{i}+r_{k}+b_{kl}+e_{ikl},$$

where $y_{ikl}$ is the observation of the *i*th genotype in the *l*th block of the *k*th replicate, μ is the general intercept, $g_{i}$ is the effect of the *i*th genotype, $r_{k}$ is the effect of the *k*th replicate, $b_{kl}$ is the effect of the *l*th block within the *k*th replicate and $e_{ikl}$ is the residual. To obtain the best linear unbiased estimators (BLUEs), $g_{i}$ was modeled as fixed effect and all other effects as random. To assess the repeatability, the model was fitted once again with $g_{i}$ as random. And the repeatability was estimated as $H_{p}^{2}=\sigma_{gp}^{2}\;/\left(\sigma_{gp}^{2}+\frac{\sigma_{e}^{2}}{n_{r}}\right)$, $H_{e}^{2}=\sigma_{ge}^{2}\;/\left(\sigma_{ge}^{2}+\frac{\sigma_{e}^{2}}{n_{r}}\right)$, where $\sigma_{gp}^{2}$ and $\sigma_{ge}^{2}$ are the genotypic variance component for PGR and elite cultivars, respectively, and $n_{r}$ is the number of replicates, $\sigma_{e}^{2}$ is the residuals.

We estimate the broad‐sense heritability across environments by using the following LMM:

(2) $$\begin{array}{ccc} y_{ijkl} & = & \mu +E_{j}+\chi_{i}\left[g_{i}^{p}+{\left(gE\right)}_{ij}^{p}\right]+\left(1-\chi_{i}\right)\left[g_{i}^{e}+{\left(gE\right)}_{ij}^{e}\right]\\ & & +\;r_{kj}+b_{lkj}+e_{ijkl}, \end{array}$$

where $y_{ijkl}$ is the observation of the *i*th genotype in the *l*th block of the *k*th replicate in the *j*th environment, μ is the common intercept, $E_{j}$ is the effect of the *j*th environment, $\chi_{i}$ is a dummy variable taking the value 1 if the *i*th genotype is a PGR, and 0 if it is an elite cultivar, the effect of the *i*th genotype is denoted by $\;g_{i}^{p}$ (if it is a PGR) or $g_{i}^{e}$ (if it is an elite cultivar), the interaction effect between the *i*th genotype and the *j*th environment is denoted by $gE_{i}^{p}$ (PGR) or $gE_{i}^{e}$ (elite cultivar), $r_{kj}$ is the effect of the *k*th replicate in the *j*th environment, $b_{lkj}$ is the effect of the *l*th block within the *k*th replicate in the *j*th environment and $e_{ijkl}$ is the residual. All effects except the intercept were assumed to be random.

The broad sense heritability across environments for the PGR ($H_{p}^{2}$) and the elite cultivars ($H_{e}^{2}$) was estimated separately as following:

$$H_{p}^{2}=\sigma_{gp}^{2}\;/\left(\sigma_{gp}^{2}+\frac{\sigma_{gEp}^{2}}{n_{\mathrm{Env}}}+\frac{\sigma_{e}^{2}}{n_{r}\;\times \;n_{\mathrm{Env}}}\right),$$

(3) $$H_{e}^{2}=\sigma_{ge}^{2}\;/\left(\sigma_{ge}^{2}+\frac{\sigma_{gEe}^{2}}{n_{\mathrm{Env}}}+\frac{\sigma_{e}^{2}}{n_{r}\;\times \;n_{\mathrm{Env}}}\right),$$

where $\sigma_{gp}^{2}$ and $\sigma_{ge}^{2}$ are the genotypic variance component for PGR and elite cultivars, respectively, $\sigma_{gEp}^{2}$ and $\sigma_{gEe}^{2}$ are the variance component of genotype‐by‐environment (G × E) interaction for PGR and elite cultivars, respectively. $n_{\mathrm{Env}}$ is the number of environments ($n_{\mathrm{Env}}=4$) and $n_{r}$ is the number of replicates ($n_{r}=2$).

### 2.3 Genomic data analysis

Genotyping of the wheat core collection was conducted using whole‐genome resequencing (WGS). Detailed procedures for library preparation, sequencing, and variant calling have been described in previous research (Schulthess, Kale, Zhao, et al., 2022). High‐quality sequencing reads were aligned to the bread wheat reference genome v2.1 of Chinese Spring (Zhu et al., 2021). Quality filtering and data management were performed using PLINK v1.90 (Chang et al., 2015), VCFtools v0.1.16 (Danecek et al., 2011), and BCFtools v1.15.1 (Danecek et al., 2021). The genotyping dataset was filtered to retain single‐nucleotide polymorphisms (SNPs) with a quality score (QUAL) ≥ 40, missing rate per individual ≤ 30%, heterozygosity ≤ 0.01, and minor allele frequency (MAF) ≥ 0.05. Read depth thresholds were set to ≥ 1 for homozygous calls and ≥ 2 for heterozygous calls. Only bi‐allelic markers were kept. Missing genotype data were imputed using BEAGLE (version 5.2) (Browning & Browning, 2009). After filtering, 24,323,883 high‐quality SNPs were retained for PGR and 9,384,031 for elite cultivars.

To elucidate the phylogenetic relationships, a neighbor‐joining tree was constructed using p‐distance calculated with VCF2Dis v1.50 (Xu et al., 2025) and visualized in iTOL (https://itol.embl.de). Linkage disequilibrium (LD, measured as 𝑟^2^) was analyzed using PopLDdecay v3.43 (C. Zhang et al., 2019). Nucleotide diversity (*𝜋*) was estimated using VCFtools v0.1.16 (Danecek et al., 2011) in 500‐kb sliding windows with a 200‐kb step size.

For the genetic analysis of Pgt samples, urediniospores of Pgt were collected from infected host leaves (Table S3) and separately propagated on the wheat cultivar Triso (*Triticum aestivum* L.) (Nedelkou et al., 2017), using the propagation method described by Patpour et al. (2022). Details of procedures for DNA extraction, library preparation, sequencing, variant calling, and genetic analyses are described in the Supporting Information. In brief, DNA from propagated urediniospores of each Pgt sample was extracted using a modified protocol from previous research (Schwessinger & Rathjen, 2017) based on CTAB and phenol‐chloroform. Samples were sequenced by Illumina short‐read technology at an average sequencing depth of 16× coverage relative to genome assembly KSU_Pgt_99KS76A_2.0 (Guo et al., 2022), which was used as reference for read mapping and variant calling. The collection was analyzed for underlying genetic structure using a filtered set of 76,026 bi‐allelic, polymorphic SNPs. The optimal number of clusters for the data was determined by a combination of heuristic methods and the gap statistic. The K‐means method was used to group samples into clusters. To assess genetic relationships independently of centroid‐based approaches, hierarchical clustering applying the unweighted pair group method with arithmetic mean was used on the pairwise genetic distances between samples, which were based on bitwise Euclidean metrics.

### 2.4 Genome‐wide association mapping

In this study, we applied a systematic genome‐wide association study (GWAS) approach to test marker effects within and across environments, as well as the marker‐by‐environment interaction effects (J. Wang, personal communcation, 2025). We adjusted and simplified the model to fit the data set in this study. The model is briefly described as follows.

First, we tested the main additive effect and the additive‐by‐environment interaction effect for each marker by using the following model:

(4) $$y=1_{nk}\mu +1_{k}⊗ma+\left[\begin{array}{c} I_{k-1}⊗m\\ 0_{n\times \left(k-1\right)} \end{array}\right]a_{e}+Z_{1}e+Z_{2}g+i+\varepsilon ,\;$$

where $y=(y_{11},\;y_{21},\;\text{…}\;y_{n1},\;y_{12},\;y_{22},\;\text{…}\;y_{n2},\;\text{…},y_{1k},\;y_{2k},\;\text{…}\;y_{nk})$ is an nk‐dimensional vector of phenotypic observations, *n* is the number of genotypes and *k* is the number of environments, $y_{ij}$ denotes the BLUEs of the *i*th genotype in the *j*th environment; $1_{nk}$ is the nk‐dimensional vector of ones, $0_{n\times (k-1)}$ is the n×(k−1) matrix of zeros, μ is the common intercept; a is the additive effect of the marker being tested (modeled as fixed effect), m is the *n*‐dimensional coding vectors of the marker, ⊗ denotes the tensor product of two matrices (vectors are treated as matrices with one column). e is the *k*‐dimensional vector of environmental effects, $\;e∼N(0,\Omega \sigma_{e}^{2})$, Ω is the environmental covariance matrix estimated by the phenotypic correlation matrix among environments (Martini et al., 2020). $Z_{1}=I_{k}\;⊗1_{n}$ is the design matrix allocating each record in y to the corresponding environment; $a_{e}$ is the (*k* −1)‐dimensional vector whose entries are the interaction effect between the marker and the first (k−1) environments. The interaction effect for the last environment is not modeled because of collinearity; g is the *n*‐dimensional vector of total additive genetic effects, $g∼N(0,G\sigma_{g}^{2})$, where G is the additive genomic kinship matrices calculated as $G=MM^{\prime }\;/c$, M is the coding matrix of all markers and $c=\mathrm{mean}(\mathrm{diag}\left(MM^{\prime }\right)$ (Vitezica et al., 2017), $Z_{2}=1_{k}\;⊗I_{n}$ is the design matrix allocating each record in y to the corresponding genotype. i is the *nk*‐dimensional vector of interaction effects between the total additive genetic effects and the environments, $\;i_{a}\;∼N\left(0,\left(\Omega ⊗G\right)\sigma_{a\times e}^{2}\right)$. 𝜀 is the *nk*‐dimensional vector of residual effects, $\varepsilon ∼N(0,I_{nk}\sigma_{\varepsilon }^{2})$. The Wald test was applied to evaluate the significance of the estimated main effect $\hat{a}$ and interaction effects ${\hat{a}}_{e}$. Specifically, the test statistics are $T_{a}={\hat{a}}^{2}\;/\mathrm{var}\left(\hat{a}\right)$ and 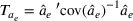. Both follow the $\chi^{2}$‐distribution, while the former has one degree of freedom and the latter (k−1) degrees of freedom.

Then, we tested the additive effects of all markers within each environment by using the following model, which is only slightly different from the first one:

(5) $$y=1_{nk}\mu +\left(I_{k}⊗m\right)a_{s}+Z_{1}e+Z_{2}g_{a}+i_{a}+\varepsilon ,\;$$

where all notations are the same as in Equation (4) except that $a_{s}$ is the *k*‐dimensional vector of within‐environment additive effects of the marker being tested. Namely, 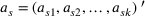 and for each i∈{1,2,…,k}, $a_{si}$ is the additive effect of the marker in the *i*th environment. Similarly, the Wald test was applied to test the significance of each estimated effect ${\hat{a}}_{si}$. The test statistic is $T_{a_{si}}={\hat{a}}_{si}^{2}\;/\mathrm{var}\left({\hat{a}}_{si}\right)$, following a $\chi^{2}$‐distribution of one degree of freedom.

The two GWAS models were implemented by inhouse‐developed R scripts. We followed the so‐called P3D approach (Z. Zhang et al., 2010) in which all variance components were estimated only once in the model without including any marker and fixed throughout the test for all markers. The genome‐wide threshold for the *p* values was determined as *p* < 0.05 after Bonferroni correction for multiple testing (Dunn, 1961). All LMMs in this study were implemented using ASReml‐R version 4.2 (Gilmour et al., 1995).

### 2.5 QTL grouping and gene annotation

LD was calculated among all marker‐trait associations (MTAs). SNPs located on different chromosomes but showing high LD (*r* ^2^ > 0.7) were considered potential misassignments and were excluded from subsequent analyses. Additionally, MTAs assigned to unknown chromosomes that exhibited strong LD with QTL regions on any of the 21 wheat chromosomes were regarded as redundant and thus removed. MTAs from all test models were grouped into QTL based on pairwise LD using the clump function in PLINK v1.9 (Chang et al., 2015). MTAs located on the same chromosome and within a 50 Mbp physical window were assigned to the same QTL if their LD, measured as *r* ^2^, exceeded 0.3. The phenotypic variance explained (PVE) by each QTL, represented by its most significant lead SNP, was estimated using linear regression models implemented in the R environment (v4.1.1; R Core Team, 2021).

All annotated high‐confidence genes mapping within the intervals of the eight QTL detected in our study were extracted from the Chinese Spring reference genome (v2.1) (Zhu et al., 2021). Gene function annotation was downloaded from eggNOG‐mapper (http://eggnog‐mapper.embl.de) and Triticeae‐Gene Tribe (http://wheat.cau.edu.cn/TGT/). The list of confirmed *NLR* genes included 1827 gene models encompassing a P‐loop, a minimum of three consecutive motifs of the NB‐ARC domain, and one or more motifs associated with LRRs (Steuernagel et al., 2020). ANNOVAR (K. Wang et al., 2010) was used to annotate sequence variants within gene regions, including untranslated regions, exons, and introns. Furthermore, variants were classified according to their location: those within 2 kb upstream of the transcription start site or downstream of the transcription termination site were distinguished from variants in the remaining intergenic regions.

### 2.6 Mining potential novel loci from PGR

To identify loci relevant to SR resistance originating from PGR but potentially novel in the elite group, we first compared the favorable (lower susceptibility) allele frequency (FAF) of all 1942 MTAs between the PGR population and the elite varieties. However, it is insufficient to look at the FAF of the MTAs because there exist non‐significant SNPs in high LD with the MTAs, and their FAF may be different from the FAF of the MTAs themselves. Therefore, all SNPs in high LD (*r* ^2^ ≥ 0.6) with the 1942 MTAs within a 50 Mbp window were clustered using the clump function in PLINK v1.9 (Chang et al., 2015). The FAF of SNPs in each cluster was then examined across 49 elite accessions. Clusters were considered novel if at least 90% of their SNPs had FAF ≤ 0.05 in the elite varieties.

To evaluate the PVE contributed by each of the five novel clusters (see Section 3.4 for details of the results) in addition to all QTL represented by their lead SNPs, the following procedure was implemented. For each novel cluster, the most significant SNP was picked out as the representative MTA. Two linear regression models were compared: (1) the full model, which included the lead SNPs of all QTLs and the representative MTA of each of the novel cluster under evaluation; and (2) the reduced model, which excluded the representative MTA from the full model. If the representative MTA of the novel cluster happened to be the lead SNP of a QTL, then the full model just included the lead SNPs of all QTL, and the reduced model was obtained by excluding the representative SNP. The difference in total *R* ^2^ explained by all variables between the full and reduced models was defined as the additional PVE specifically contributed by the novel cluster. We also estimated the PVE of the five representative MTAs together by a similar approach. In this case, the full model included the lead SNPs of all QTL and the representative MTAs of all five novel clusters, and the reduced model was obtained by excluding the lead SNPs of all QTLs, except those identical to the representative MTAs.

## 3 RESULTS

### 3.1 Molecular diversity of the underlying wheat population

Whole‐genome sequencing of the 250 genotypes was performed using the Illumina NovaSeq 6000 system (Schulthess, Kale, Zhao, et al., 2022), achieving an average genome coverage of threefold. The sequencing reads were mapped to the wheat reference genome v2.1 of Chinese Spring (Zhu et al., 2021), resulting in 24,323,883 high‐quality SNPs for the PGR population and 9,384,031 for the elite varieties after quality control. SNPs with an MAF ≥ 0.05 and a missing rate ≤ 0.3 across the 250 genotypes were retained (Figures S1 and 2). To ensure sample consistency, the WGS data were compared with previously generated genotyping‐by‐sequencing data (Schulthess, Kale, Zhao, et al., 2022). Of the 250 genotypes, 243 (49 elite varieties and 194 PGR) showed concordance and were included in the subsequent analyses.

**FIGURE 1:**
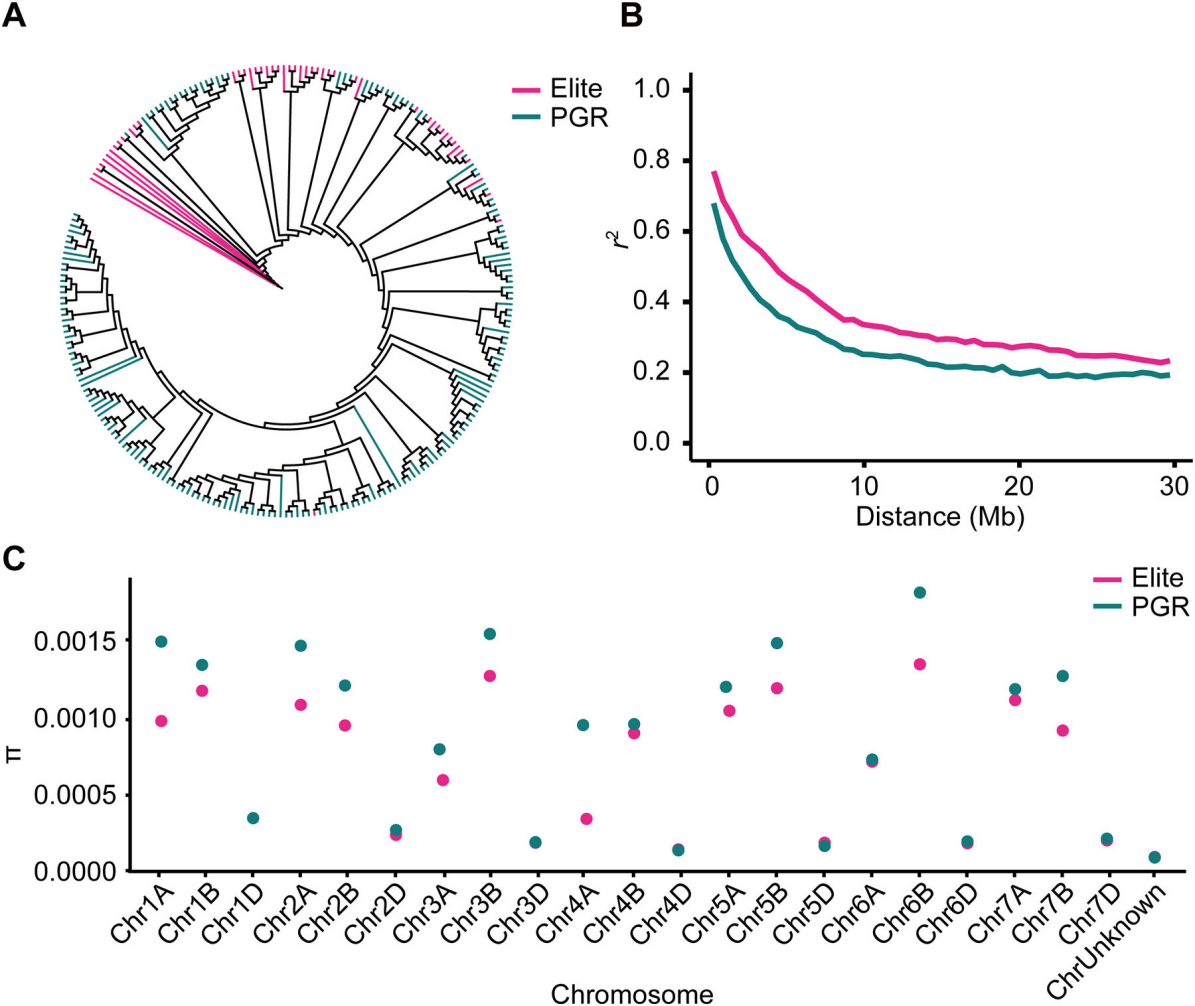
Population structure and diversity. (A) Neighbor‐joining tree of 243 wheat genotypes (194 plant genetic resources [PGR]; 49 European elite varieties) constructed using whole‐genome single‐nucleotide polymorphisms (SNPs). (B) Linkage disequilibrium (LD) decay patterns in PGR and elite populations. (C) Nucleotide diversity (*π*) of the two panels.

To investigate the genetic relationships among the 243 samples, we constructed a phylogenetic tree (Figure 1A). The analysis revealed that elite lines tended to cluster separately from the PGR, though there were exceptions. This can be attributed to their shared European origin. Next, we assessed LD decay and observed that it declined more rapidly in the PGR group than in the elite group (Figure 1B). This suggests greater genetic diversity in the PGR population, which was consistent with the higher nucleotide diversity observed in the PGR accessions across most chromosomes (Figure 1C).

### 3.2 Genotype‐environment interactions persisted despite controlled inoculation

**FIGURE 2:**
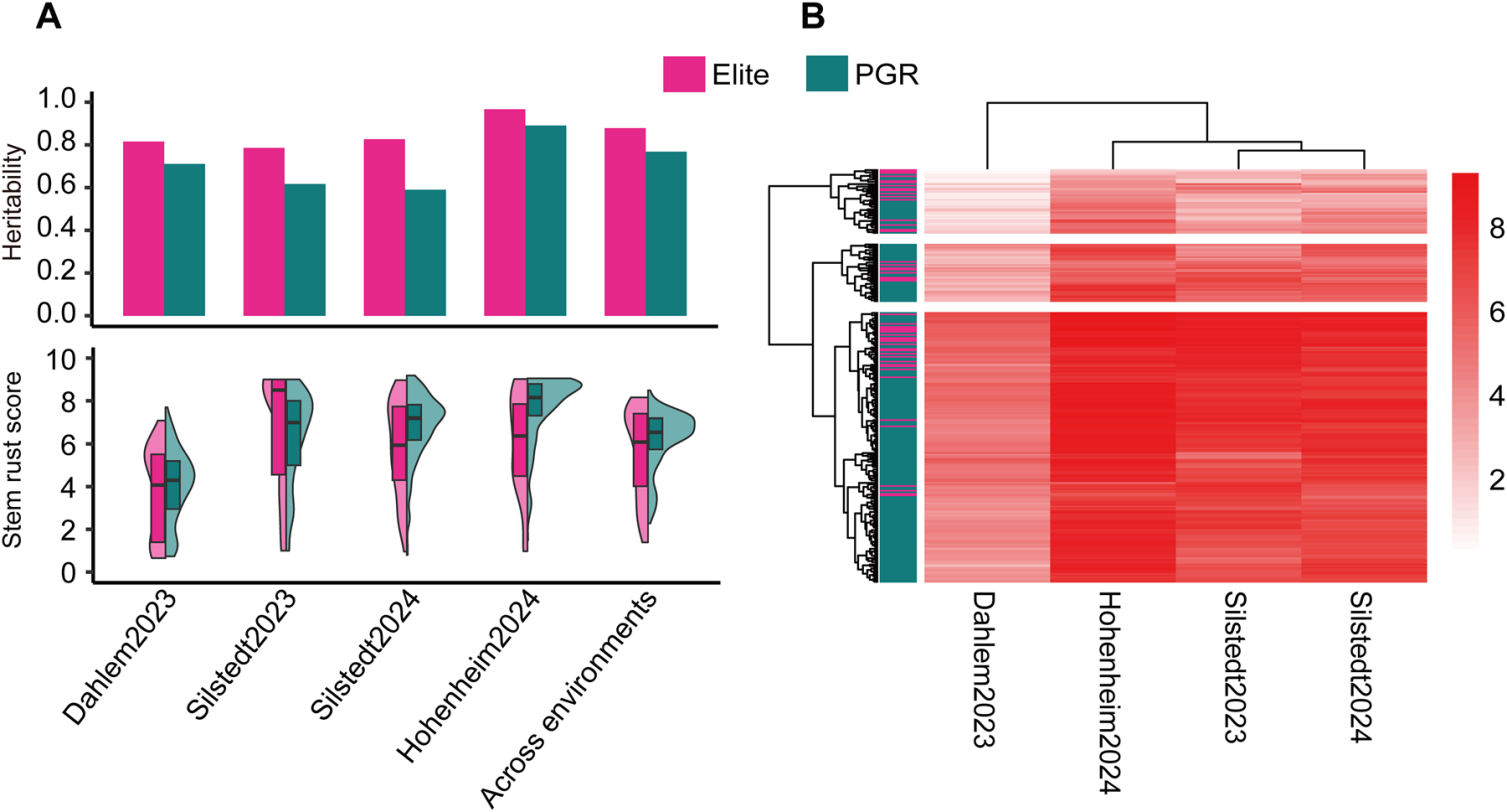
Phenotypic variation and heritability of stem rust resistance of a population of 200 plant genetic resources (PGR) and 50 elite lines. (A) Heritability (top) and phenotypic distribution (bottom) of the best linear unbiased estimates (BLUEs) of stem rust (SR) severity in each environment and across environments for the elite and PGR groups. The scale of the SR score was between one (the most resistant) and nine (the most susceptible). (B) Heatmap showing genotype‐by‐environment interaction effects for SR resistance predicted within individual environments. Rows represent genotypes and columns represent environments. Light red colors indicate stronger resistance. Hierarchical clustering was applied to genotypes and environments.

We evaluated the 250 genotypes for their response to SR across four environments using artificial inoculation with a mix of pathogen races. In all environments, we observed that disease pressure facilitated genetic differentiation, resulting in significant genotypic variances (*p* < 0.001) and heritability estimates of ≥ 0.6 within environments for the elite and PGR groups (Figure 2A). Analyses across environments yielded broad‐sense heritability estimates of 0.88 for the elite group and 0.77 for the PGR group. The variance components of the G × E interaction effects were significantly (*p* < 0.001) greater than zero (Table S4). This prompted us to examine the G × E pattern in greater detail. Our analysis revealed an absence of pronounced clusters of environments or differences between the elite and PGR groups (Figure 2B).

**FIGURE 3:**
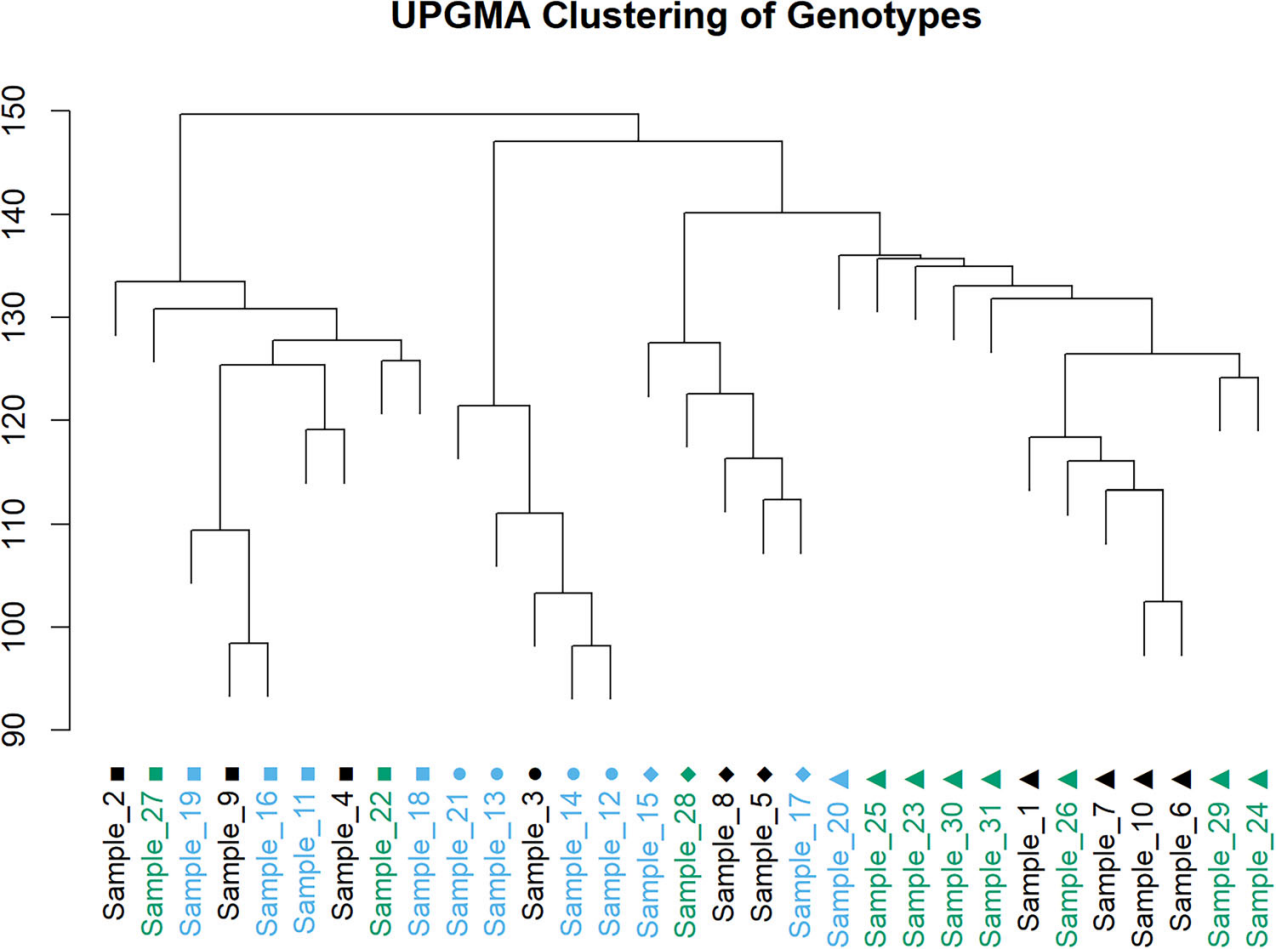
Unweighted pair group method with arithmetic mean (UPGMA) dendrogram and K‐means clustering of *Puccinia graminis* f. sp*. tritici* samples. Hierarchical clustering and K‐means clustering of thirty‐one *Puccinia graminis* f. sp. *tritici* samples collected in 2023 using a quality‐filtered set of 76,026 bi‐allelic, polymorphic single‐nucleotide polymorphism (SNP) were used to highlight genetic relationships and genetic structure in the collection, with closely related individuals clustering at lower heights. The unweighted pair group method with arithmetic mean was used on pairwise genetic distances between samples, which were computed using bitwise Euclidean metrics. The *y*‐axis represents the square root of the number of differing SNPs between samples or clusters. The K‐means method with cluster number set to *k* = 4 was used to group samples into clusters. Sample labels are color‐coded by collection location (Dahlem—black; Hohenheim—green; Silstedt—blue), and cluster assignments are appended as symbols to each label.

Whole‐genome Illumina sequencing (16× genome coverage) of Pgt samples collected from all locations in 2023 (Table S3) was applied to gain first insights into whether the local pedoclimatic conditions impacted the genetics of the Pgt inoculum. For each sample over 95% of sequenced reads aligned with mapping quality scores ≥ 30 against the reference genome assembly. Clustering analysis on 76,026 filtered SNPs by the silhouette method determined *k* = 4 as optimal number of clusters, while the elbow method and gap statistic determined k to be three. Despite this number of clusters and Hamming distances ranging from 9,604 to 27,225 pairwise SNP differences between individual samples (12.6%–35.8% divergence), clustering of samples into *k* = 4 clusters indicated that the genetic structure in the data are not congruent with the sample's locations (Figure 3). All four genetic clusters included representatives from at least two of the three sampling locations.

### 3.3 Genome‐wide association analyses for SR stress responses in the PGR group

**FIGURE 4:**
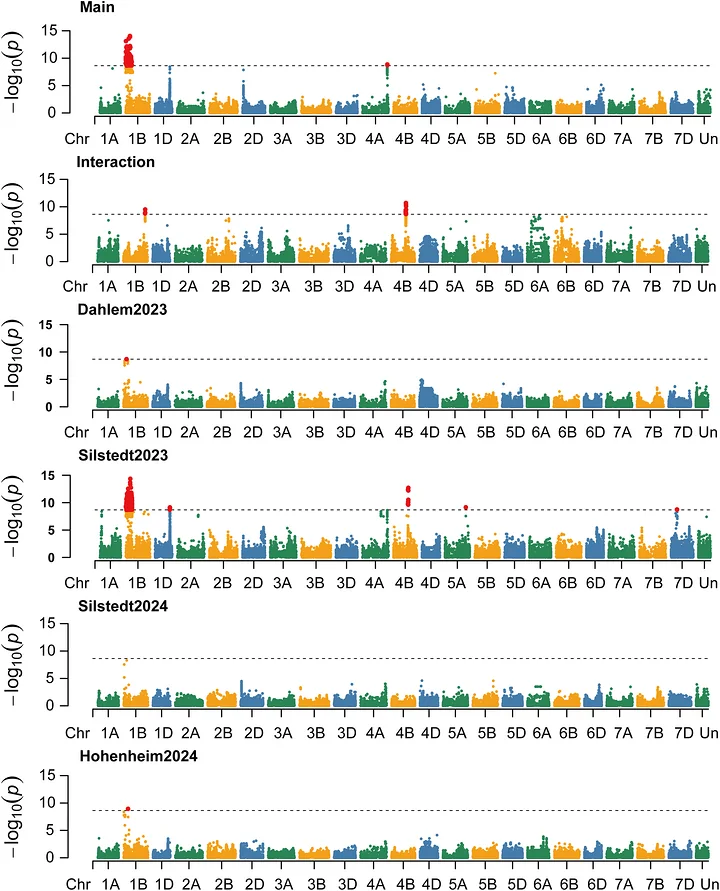
Genome‐wide association study results for stem rust resistance across four environments. Manhattan plots are shown for the main effects, marker‐by‐environment interactions, and environment‐specific effects. The gray horizontal dashed line indicates the genome‐wide significance threshold (−log_10_(*p*) = 8.69) after Bonferroni correction.

We performed genome‐wide association mapping across four environments using field data from the PGR population. We used a multi‐environment GWAS model for the analysis. This model considered main effects of marker across environments, SNP‐by‐environment interaction (SNP×E) effects, and environment‐specific marker effects. The analysis identified 637 significant MTAs (−log_10_(*p*) ≥ 8.69) on chromosome 1B (635) and 4A (2) for main effects (Figure 4; Table S5). Notably, none of the 637 MTAs exhibited significant interactions with the environments. Despite this, 22 significant SNP×E effects were detected on chromosome 1B (3) and 4B (19). For five (4B) out of the 22 SNP×E interaction effects, MTAs were detected in the analyses of individual environments. In addition to the 637 MTAs detected for main effects, we identified 1291 environment‐specific MTAs in single‐environment analyses that were not captured in the main‐effect analyses, resulting in the total number of MTAs being 1942.

The MTAs were grouped into QTL regions using an LD threshold of *r* ^2^ ≥ 0.3, and QTL were designated by chromosome number followed by their physical start and end positions (Mbp). Eight QTL were identified across chromosomes 1B, 1D, 4A, 4B, 5A, and 7D (Figure S3, Table S5). The QTL *QSr1B_88.99_178.52* (on chromosome 1B, spanning 88.99 Mbp to 178.52 Mbp) was consistently detected in two environments, as well as in the main‐effect test. One QTL showed significant QTL×E interaction effects. *QSr4A_716.31_716.32* was only detected in the main‐effect test. The PVE for each QTL was estimated based on the SNP with the strongest association, as indicated by the smallest *p*‐value in each environment. The proportion of genotypic variance explained by individual QTL ranged from 2.29% to 13.30% in main‐effect analysis and from 0.84% to 28.19% in single‐environment analyses. In the environment Silstedt2023, the majority of QTL explained a considerable portion of phenotypic variance, ranging from 12.09% to 23.82%. QTL *QSr1B_1.47_67.79*, *QSr1B_88.99_178.52*, and *QSr1B_178.54_222.33* consistently explained the highest proportion of the explained genotypic variance across all tested environments.

### 3.4 Identification of novel resistant loci for SR from PGR

**FIGURE 5:**
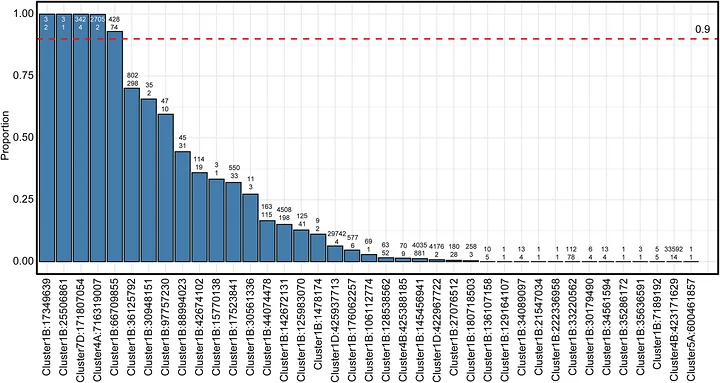
Proportion of single‐nucleotide polymorphisms (SNPs) with favorable allele frequencies (FAF) ≤ 0.05 in the elite group for 39 clusters. In each bar, the first number indicates the total number of markers within the cluster, and the second number indicates the number of marker‐trait associations. The red dashed line indicates the threshold (FAF = 0.9) for claiming novel clusters.

To identify potentially novel sources of resistance to SR from the PGR population that are absent in the elite pool, we calculated the favorable (lower susceptibility) allele frequency of the 1942 MTAs in both the PGR and elite groups. The FAF of these MTAs ranged from 0 to 0.49 in the elite group and from 0.06 to 0.61 in the PGR group (Figure S4; Table S5). According to the FAF in the elite group, 328 MTAs were classified as putative novel (FAF ≤ 0.05). The favorable allele of 88 MTAs was completely absent in the elite lines (FAF = 0). However, there were non‐significant SNPs in high LD with these MTAs, and their favorable allele may not be rare in the elite varieties. Therefore, we examined the FAF of all SNPs, including the non‐significant ones, in high LD with all MTAs. These SNPs were assigned to 39 clusters by using an LD threshold of *r* ^2^ ≥ 0.6 (Figure 5). Each cluster contained at least one MTA. The clusters were named after their representative MTAs, that is, the most significant MTAs within the cluster. Among the 39 clusters, five were classified as putative novel, following the criterion that at least 90% of the SNPs in a cluster had a FAF smaller than 0.05 in the elite group (Table 1).

**TABLE 1:** Information of the five novel clusters in which the favorable allele frequency (FAF) of at least 90% of the single‐nucleotide polymorphisms (SNPs) was <0.05 in the elite group.

Novel cluster	QTL	No. of SNPs	No. of MTAs	Rep. MTA^a^	FAF of Rep. MTA (PGR)	FAF of Rep. MTA (Elite)	FAF ≤ 0.05^b^ SNPs (Elite)	PVE^c^ main^d^	PVE E1^e^	PVE E2^f^	PVE E3^g^	PVE E4^h^
Cluster1B:17349639	*QSr1B_1.47_67.79*	3	2	Chr1B:17349639	0.21	0	100.00%	0.57%	0.82%	0.80%	1.21%	0.01%
Cluster1B:25506861	*QSr1B_1.47_67.79*	3	1	Chr1B:25506861	0.14	0	100.00%	0.01%	0.87%	0.52%	0.30%	0.01%
Cluster1B:66709855	*QSr1B_1.47_67.79*	428	74	Chr1B:66709855	0.11	0	92.99%	0.35%	0.39%	0.48%	0.25%	0.06%
Cluster4A:716319007	*QSr4A_716.31_716.32*	2705	2	Chr4A:716319007	0.09	0.02	100.00%	1.14%	1.30%	0.02%	2.96%	0.43%
Cluster7D:171807054	*QSr7D_171.80_174.34*	342	4	Chr7D:171807054	0.1	0.02	100.00%	1.86%	1.22%	1.43%	0.01%	1.19%

^a^ Rep. Marker‐trait associations (MTA): The most significant SNP was defined as the representative MTA (Rep. MTA) within the cluster.

^b^ The proportion of SNPs whose FAF ≤ 0.05 within the cluster for the Elite population.

^c^ PVE: the proportion of phenotypic variance explained by the Rep. MTA. Here, we provided a conservative estimate which exclude the contribution of lead SNPs of all quantitative trait loci (QTL).

^d^ PVE main: the PVE of the main effect of the Rep. MTA across environments.

^e–h^ PVE E1–E4: the PVE of the Rep. MTA in each environment. E1, Dahlem2023; E2, Silstedt2023; E3, Silstedt2024; E4, Hohenheim2024.

**FIGURE 6:**
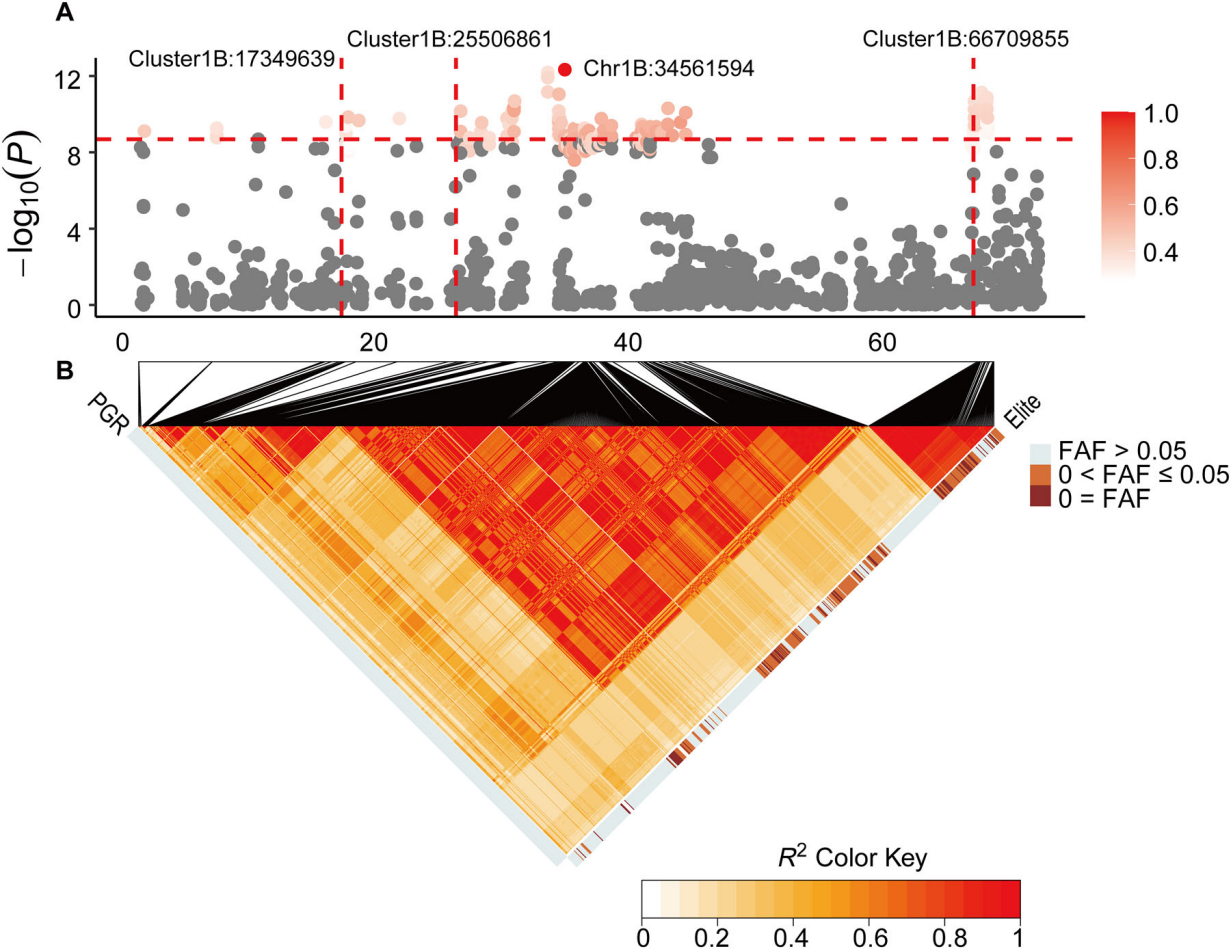
Detailed information of a quantitative trait loci (QTL) for stem rust resistance on chromosome 1B under main effect. (A) Manhattan plot for all markers in the QTL *QSr1B_1.47_67.79* (1.47–67.79 Mbp). The dashed horizontal line indicates the genome‐wide significance threshold (−log_10_(*p*) = 8.69). The lead (most significant) single‐nucleotide polymorphisms (SNP) was colored red. The color of other significant SNPs was defined according to its linkage disequilibrium (LD) with the lead SNP. Non‐significant SNPs were colored gray. The position of representative SNPs of three novel clusters were indicated by red dashed vertical lines. (B) The heatmap of LD among the 706 significant SNPs within the quantitative trait loci (QTL) region. Favorable allele frequencies (FAF) of significant markers were indicated as colored bars on the left (FAF in the plant genetic resources [PGR] panel) and on the right (FAF in the elite panel).

Two novel clusters, Cluster4A:716319007 and Cluster7D:171807054, were located in QTL *QSr4A_716.31_716.32* and *QSr7D_171.80_174.34*, respectively. The LD structure of these two QTL was simple, as each had only one cluster. Three novel clusters (Cluster1B:17349639, Cluster1B:25506861, and Cluster1B:66709855) were located in the QTL *QSr1B_1.47_67.79*. Note that the FAF of the lead SNP in this QTL was 0.18 in the elite group. Thus, focusing on the lead SNP would not reveal any novel loci. In fact, the lead SNP exhibited moderate LD (*r* ^2^ = 0.16–0.67) with other MTAs over a wide range, suggesting a complex LD structure in the region (Figure 6). In this QTL, there were 273 MTAs that had an FAF of <0.05 in the elite group. These MTAs belonged to 21 clusters, but only three of them were novel. Our results indicate the importance of checking the FAF of all SNPs in high LD with the MTAs for QTL regions with a complex LD structure.

**FIGURE 7:**
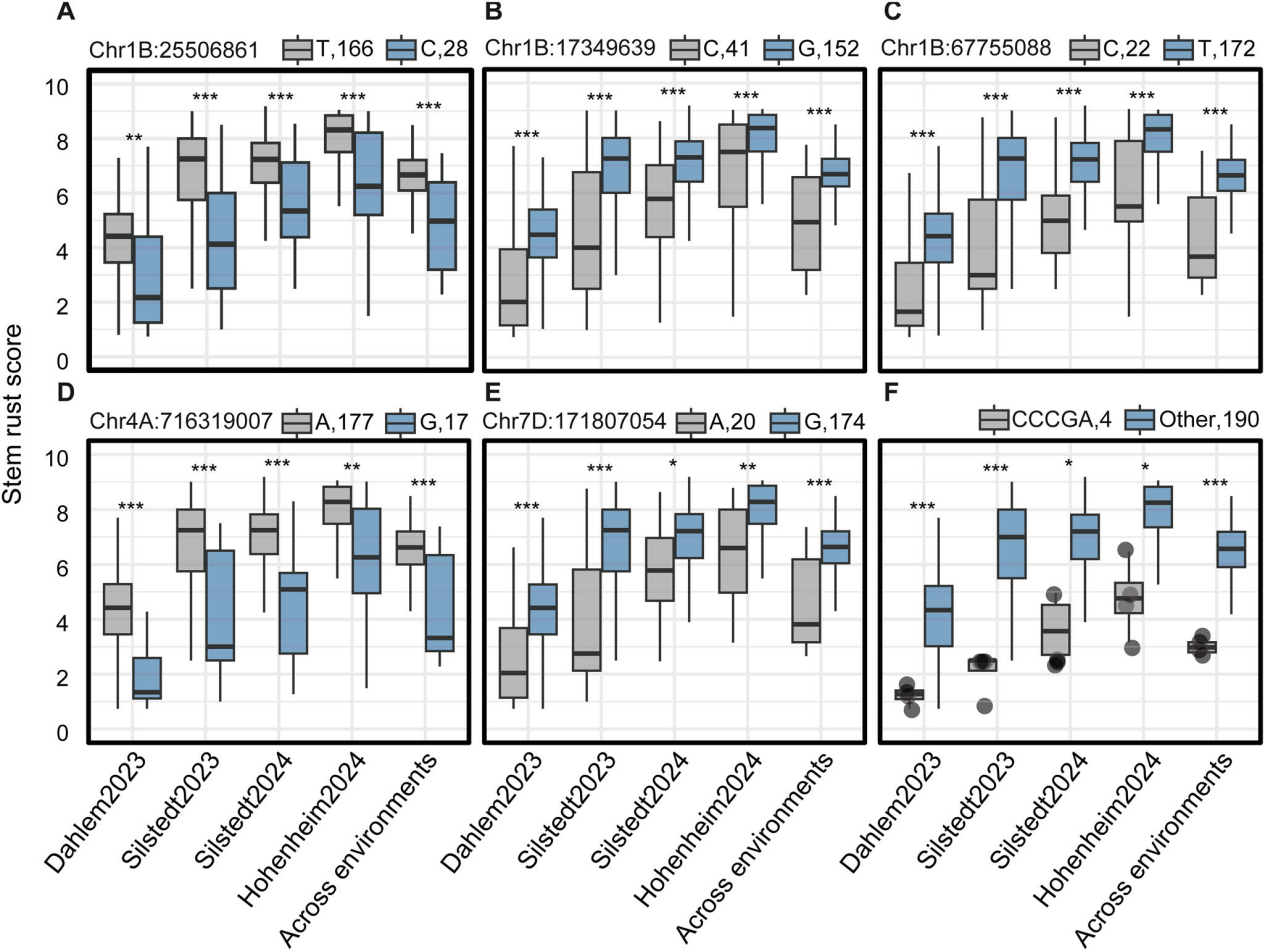
Phenotypic distribution of 194 plant genetic resources carrying favorable and unfavorable alleles. (A—E) Each boxplot showed the phenotypic distribution of the accessions carrying favorable and unfavorable alleles for one of the five representative markers of novel clusters. F The phenotypic distribution of four genotypes carrying all five favorable alleles compared to other genotypes. Statistical significance was determined using two‐tailed Student's *t*‐tests. Significance levels are indicated by stars: ***p < 0.001, **p < 0.01, *p < 0.05, and NS (non‐significant).

We applied a conservative approach to estimate the proportion of PVE by the five novel clusters, in addition to the lead SNPs of all QTL (see Section 2.6 for details of the method). The estimated PVE was small for each of the five representative MTAs (Chr1B:25506861, Chr1B:17349639, Chr1B:66709855, Chr4A:716319007, Chr7D:171807054; Table 1). However, the PVE of the five MTAs together amounted to 2.40%–7.03%, depending on the environment. Genotypes carrying favorable alleles at these MTAs exhibited significantly improved resistance to SR (Figure 7). Four genotypes from the PGR population carrying all five favorable alleles were selected as potential donors for future breeding to enhance resistance (Figure 7).

## 4 DISCUSSION

### 4.1 Integrating G × E interactions in the study of SR stress responses

Recent epidemics have highlighted the increased risk of SR outbreaks in Europe due to rising temperatures (Olivera Firpo et al., 2017). This underscores the importance of identifying potential sources of SR resistance for European wheat breeding (Miedaner et al., 2024). However, testing for SR resistance in the field is complicated by the significant impact of environmental factors and their interactions with the genotypes (Kemboi et al., 2025). To address this challenge, we phenotyped 200 PGR and 50 elite lines for SR resistance in multi‐environment field trials that were artificially inoculated with a mixture of SR races. Despite the inoculation, statistical analyses of the phenotypic data revealed that SR susceptibility was significantly influenced by the environment, resulting in significant G × E interactions (Table S4). These findings are consistent with those reported by Kemboi et al. (2025), who evaluated 25 spring wheat lines from the CIMMYT nursery at the international SR phenotyping platform in Kenya under artificial inoculation. Their study demonstrated significant effects of season as well as genotype × season interaction on the area under the disease progress curve (AUDPC). Our findings are also in line with a recent study that performed QTL mapping in multiple crosses between European elite lines (Miedaner et al., 2024). This study also found pronounced variance in environmental and G × E interaction effects for SR resistance in artificially inoculated field trials.

In light of the significant G × E interactions, we accounted for them in the association mapping study. Most QTL were detected in only one environment, and G × E interactions contributed to the observed variation explained by QTL within each environment (Figure 4; Figure S3). For example, the variation explained by QTL *QSr4B_413.24_425.52* ranged from 3.73% in Dahlem2023 to 18.81% in Silstedt2023. These pronounced differences are surprising, considering that the same mixed Pgt inoculum was applied across all environments, and sequence‐based analyses revealed no significant divergence in the overall pathogen population composition between locations (Figure 3). Therefore, we speculated that local pedoclimatic factors, such as temperature, drought, and humidity, may have shaped the interactions between Pgt and individual wheat genotypes, leading to G × E interactions. Taking temperature as an example, we found that optimal infection temperature has been identified as an important contributor to Pgt occurrence (Flath et al., 2018). On the other hand, temperature‐sensitive immunity was reported for resistance genes *Sr6*, *Sr13*, and *Sr21* (Hewitt et al., 2025). Consequently, incorporating specific environmental data alongside a plot‐based sequencing strategy of Pgt composition could provide a more thorough understanding of the genetic underpinnings and occurrence patterns of SR resistance.

### 4.2 Comparison of detected QTL with previous findings

The 1942 (−log_10_(*p*) ≥ 8.69) MTAs identified in our study were grouped based on their local LD into eight QTL (Figure S3). Five of the QTL (Table S6) overlapped with loci that had been reported previously (Cheng et al., 2024; Marone et al., 2022; Mastrangelo et al., 2025; Miedaner et al., 2024; Njau et al., 2013; Tong et al., 2024). For instance, the QTL *QSr1B_1.47_67.79* on chromosome 1B co‐localized with the well‐characterized *Sr31* locus (Miedaner et al., 2024) and explained a significantly higher proportion of phenotypic variance (18.29%–31.07%) than most QTL in our study. These findings are consistent with a recent QTL mapping study in Central European wheat germplasm (Miedaner et al., 2024), suggesting that the gene remains effective following the 2013 SR outbreak (Olivera Firpo et al., 2017). Interestingly, the complexity of the 1B chromosome region is well established, and it has long been recognized that multiple variants of the *Sr31* resistance gene exist. Two primary biotypes have been described: the substitution biotype 1BL.1RS and the translocation biotype 1R(1B) (Mettin et al., 1973; Zeller, 1973). Notably, some wheat cultivars harbor both biotypes simultaneously (Zeller, 1973). Furthermore, *Sr31* has distinct genetic origins, with evidence pointing to its introduction into modern wheat germplasm through cultivars such as Imperial rye (*Secale cereale* L.) and Amigo wheat (McIntosh et al., 1995).

In our study, we identified three QTLs that have not been reported earlier (Table S6). The QTL *QSr7D_171.80_174.34*, located on chromosome 7D from 171.80 Mbp to 174.34 Mbp, was distal to previous QTLs summarized by Tong et al. (2024) and suggests that it is also a novel locus. The two QTLs *QSr1D_422.96_428.55* and *QSr4B_413.24_425.52*, were located near previously reported QTLs, though no direct overlap was observed. For instance, *QSr4B_413.25_425.52* (413.25–425.52 Mbp) was identified on chromosome 4B close to *QSr.sun‐4B* (∼381.54 Mbp). Given the potential complex LD structures, the novelty of these QTLs must be confirmed in follow‐up studies, even in the absence of overlap.

We extracted a list of genes located in the regions of the eight QTL detected in our study. We then applied gene annotation and searched for MTAs that caused stop‐gain or non‐synonymous mutations, which serve as a signal that these are putative functional polymorphisms. A total of 40 putative functional MTAs were identified on chromosome 1B, including 14 genes within *QSr1B_1.47_67.79* (one *NLR* gene, *TraesCS1B03G0068600*) and 26 within *QSr1B_88.99_178.52* (two *NLR* genes, *TraesCS1B03G0291400* and *TraesCS1B03G0303800*) (Table S7) (Steuernagel et al., 2020). *TraesCS1B03G0068600* harbors the MTA Chr1B:17349639, which corresponds to the novel cluster Cluster1B:17349639. In addition, three genes (*TraesCS1B03G0196800*, *TraesCS1B03G0197100*, and *TraesCS1B03G0197700*) were located within the novel cluster Cluster1B:66709855, characterized by a block with high LD. For QTL *QSr4A_716.31_716.32* (novel cluster Cluster4A:716319007), *TraesCS4A03G1083100*, *TraesCS4A03G1088400*, and *TraesCS4A03G1088700* were identified and annotated as disease resistance‐related genes (Table S7). No candidate genes carrying protein‐altering variants were identified in QTL *QSr1B_178.54_222.33*, *QSr1D_422.96_428.55*, *QSr4B_413.24_425.52*, and *QSr5A_600.46_600.46*.

### 4.3 Identifying SR resistance loci in PGR that are absent in the elite breeding pool

We mined for novel resistance genes present in PGR but absent in the elite wheat breeding pool by examining the FAF of markers in elite cultivars. Moreover, we considered both significant MTAs and non‐significant SNPs in high LD (*r* ^2^ ≥ 0.6) with the MTAs within a 50 Mbp range. This approach contrasts with prior strategies (Schulthess, Kale, Liu, et al., 2022), which reduced MTAs redundancy by selecting only the most significant markers per chromosome and pruning MTAs in high LD (*r* ^2^ > 0.8) or within 1 Mbp range. While effectively minimizing redundancy, such pruning may overlook linked non‐significant SNPs that, although not individually significant, could represent rare or underrepresented haplotypes in the elite pool, potentially tagging functional resistance alleles. Indeed, in our study, ∼70% of MTAs were clustered within two major regions (Cluster1B:145456941 and Cluster1B:36125792), which were not entirely novel (Figure 5). By retaining these linked variants, our method enabled the discovery of five putative novel resistance loci. We further proposed four donor lines, each carrying all five loci with additional SR resistance alleles (Figure 7), making them ideal candidates for prebreeding. Future work should focus on validating these loci in biparental populations and introgressing them into elite backgrounds through marker‐assisted selection to enhance the durability of SR resistance in modern wheat cultivars. Our approach offers a scalable, data‐driven framework for targeted discovery and deployment of novel alleles from gene banks, with broad applicability to complex traits and diverse crop species.

## AUTHOR CONTRIBUTIONS

**Jiaojiao Wang**: Data curation; formal analysis; investigation; methodology; software; visualization; writing—original draft. **Guoliang Li**: Data curation. **Tim Kloppe**: Investigation. **Kerstin Flath**: Investigation. **Philipp Schulz**: Investigation. **Mathias Wiegman**: Investigation. **Thomas Miedaner**: Investigation. **Yong Jiang**: Conceptualization; data curation; methodology; software; supervision; writing—review and editing. **Jochen C. Reif**: Conceptualization; funding acquisition; resources; supervision; writing—review and editing.

## CONFLICT OF INTEREST STATEMENT

The authors declare no conflicts of interest.

## Supporting information




**Table S1** Field trials.
**Table S2** The identities of the Puccinia graminis f. sp. tritici isolates that were used as the artificial inoculum in the field trials are listed below. The isolates were mixed in equal proportions to produce the inoculum.
**Table S3** Identities of Puccinia graminis f. sp. tritici isolates sampled from infected leaves of host plants during the 2023 evaluation period.
**Table S4** Variance components, number of environments and replications, and heritability estimates for individual and across environments.
**Table S5** Allele frequencies of 1,942 significant SNPs from all GWAS tests within Elite and PGR panels across eight QTL. The three newly identified QTL are highlighted in yellow.
**Table S6** Comparison of Identified eight QTL with previously reported QTL to identify putativelynovel Loci.
**Table S7** Variant annotation of significantly associated SNPs in four QTL. Associated SNPs (76) detected by all test are compiled.

Supplemental Materials

**FIGURE S1** Density distribution of single nucleotide polymorphisms of Elite lines. Red and gray bars represent SNP‐rich and SNP‐poor genomic regions.

**FIGURE S2** Density distribution of single nucleotide polymorphisms of plant genetic resources (PGR). Red and gray bars represent SNP‐rich and SNP‐poor genomic regions.

**FIGURE S3** Proportion of phenotypic variance explained by eight QTL across four environments and main effect. The heatmap illustrates the phenotypic variance explained by each QTL (columns) under different environments and the main effect (rows). Yellow borders highlight QTL that were statistically significant in the corresponding environments. The QTL labeled in blue indicate significant QTL‐by‐environment interactions. Parentheses indicate number of marker‐trait associations per QTL for all GWAS test.

**FIGURE S4** Favorable allele frequencies of the 1,942 marker‐trait associations in the 194 plant genetic resources and the elite lines. Marker‐trait associations in different QTL were indicated by different symbols.

## Data Availability

The genotypic datasets generated and analyzed in this study are publicly available in the European Nucleotide Archive (https://www.ebi.ac.uk/ena/) under Project IDs PRJEB48988 and PRJEB48738. Variant Call Format (VCF) files for the whole‐genome sequencing data can be accessed through the European Variation Archive (https://www.ebi.ac.uk/eva/) under Project ID PRJEB52759. All of these datasets have been previously described in Schulthess, Kale, Liu, et al. (2022). The phenotypic data for this publication will be available at e!DAL – Plant Genomics & Phenomics Research Data Repository. We confirm that the materials described in this study are available from *Leibniz Institute of Plant Genetics and Crop Plant Research (IPK)*.
