# Supplementary material for: Discovering putative novel stem rust resistance loci in wheat genetic resources
Source: Plant Genome. 2026 Jun 4;19(2):e70259. doi: 10.1002/tpg2.70259 (PMC13492613; doi:10.1002/tpg2.70259)
Supplement: Supplementary file 1 [file TPG2-19-e70259-s002.docx]

**Supplemental Information**

**Supplemental Figures**

**FIGURE S1** **Density distribution of single nucleotide polymorphisms of Elite lines.** Red and gray bars represent SNP-rich and SNP-poor genomic regions.

**FIGURE S2** **Density distribution of single nucleotide polymorphisms of plant genetic resources (PGR).** Red and gray bars represent SNP-rich and SNP-poor genomic regions.

**FIGURE S3** **Proportion of phenotypic variance explained by eight QTL across four environments and main effect.** The heatmap illustrates the phenotypic variance explained by each QTL (columns) under different environments and the main effect (rows). Yellow borders highlight QTL that were statistically significant in the corresponding environments. The QTL labeled in blue indicate significant QTL-by-environment interactions. Parentheses indicate number of marker-trait associations per QTL for all GWAS test.

**FIGURE S4 Favorable allele frequencies of the 1,942 marker-trait associations in the 194 plant genetic resources and the elite lines.** Marker-trait associations in different QTL were indicated by different symbols.

**Supplemental Methods**

**Supplemental note S1. Details on procedures for the genetic analysis of *Pgt* samples.**

**DNA extraction**

A modified protocol based on Schwessinger et al. (2017) was used for DNA extraction and sample QC with the following modifications: For each sample, 20 mg of dried, frozen spores were ground in a 2-mL screw-cap tube (Sarstedt, item number 72.693.005) loaded with one 4-mm glass bead (Roth, item number HH55.1), 50 mg of 1-mm glass beads (Sigma-Aldrich, item number Z250473) and 500 mg of quartz sand (50–70 mesh particle size; Sigma-Aldrich, item number 274739) by running 3 cycles on a Precellys Evolution homogenizer (Bertin Technologies) at 10,000 rpm for 10 s with intermittent cooling of samples in liquid nitrogen. Reaction volumes were adjusted to 2-mL tubes, such that 11,250 µL lysis buffer was used. Samples were incubated with 10 µL of a 1:1 mix of RNase A (10 mg/mL; New England Biolabs, item number T3018) and RNase T1 (1000 U/µL; Thermo Fisher Scientific, item number EN0542) at 50 °C in a rotary shaker run at 850 rpm for 1 hour for the first RNA digest, while for the second RNA digest, 5 µL of the RNase mix was used and samples were incubated following the original protocol. Each reaction was incubated with 25 µL Proteinase K (NEB, item number P8107S) in a rotary shaker run at 850 rpm for 1–2 hours (Guo et al. 2022). Samples were not enriched or purified with magnetic beads.

**Library preparation and Sequencing**

Each sample, set to 25 µL and adjusted to 12 ng/µL, was sent to the DNA Sequencing Laboratory of the Leibniz Institute of Plant Genetics and Crop Plant Research, Germany, for library preparation using the Illumina DNA Prep kit (Illumina, item number 20060059) and 150 bp paired-end sequencing on an S4 flow cell and an Illumina NovaSeq 6000 instrument, at an average read depth of 16× genome-wide coverage.

**Variant calling and filtering**

Sequence data was processed on a locally run Galaxy instance at the Julius Kuehn-Institute (The Galaxy Community et al. 2024) Reads were quality checked and trimmed on 3’ end by trimming 19 bp from the 3’ end using FastQC (Andrews 2010) and Cutadapt (Martin 2011). The trimmed reads were mapped to the genome assembly KSU_Pgt_99KS76A_2.0 (Guo et al. 2022) using BWA-MEM v0.7.17 (Li 2013), which was run at default settings and thereby filtering for mapping quality ≥ 30. Filtered alignments were marked for PCR duplicates using MarkDuplicates (Picard) in GATK v4.1.8.0 (Poplin et al., 2017). Variant call data was generated for all samples simultaneously using the filtered alignments and FreeBayes v.1.3.1 run with setting frequency-based pooled calling (Garrison et al., 2012). The joint variant call format (VCF) file was quality-filtered using BCFtools v1.15.1 (Danecek et al. 2021) for 76,026 bi-allelic polymorphic SNPs, for which all samples had a called genotype and at least 90 % of the samples had a read depth ≥ 10.

**Genetic analyses**

The collection was analyzed for underlying genetic structure in R. The R package ‘vcfR’ (Knaus et al., 2017) was used to load (function ‘read. vcfR’) and convert (function ‘vcfR2genlight’) the VCF data in R to a genlight object. With this object as input, a pairwise distance matrix was created by calculating the Euclidean Hamming distances between samples using the ‘bitwise.dist’ function in package ‘poppr’ (Kamvar et al., 2014) with the euclidean = TRUE option. Hierarchical clustering of samples based on the distance matrix was done using the unweighted pair group method with arithmetic mean (UPGMA) in function ‘hclust’ of the ‘stats’ package, which output was used to plot the dendrogram. For centroid-based clustering, sample genotypes were extracted from the vcfR object and converted to numeric dosage values representing homozygous reference, heterozygous, and homozygous alternate states using the function ‘extract.gt’ in package ‘vcfR’. The resulting genotype matrix was then used to estimate the optimal number of clusters k for the data, using the elbow and the silhouette method with function ‘fviz_nbclust’, provided in R package ‘factoextra’ (Kassambara 2017), as well as the gap statistic with function ‘clusGap’ from package ‘cluster’. The K-means method implemented in base R function ‘kmeans’ from ‘stats’ was used to group samples into clusters. The output was then superimposed on the UPGMA dendrogram using base R function ‘plot’.

**Supplemental References**

Andrews, S. (2010). A Quality Control Tool for High Throughput Sequence Data. Available online at: <http://www.bioinformatics.babraham.ac.uk/projects/fastqc/>

Danecek, P., Bonfield, J. K., Liddle, J., Marshall, J., Ohan, V., Pollard, M. O., Whitwham, A., Keane, T., McCarthy, S. A., Davies, R. M., & Li, H. (2021). Twelve years of SAMtools and BCFtools. *Gigascience*, 10(2), giab008. <https://doi.org/10.1093/gigascience/giab008>

Garrison, E., & Marth, G. (2012). Haplotype-based variant detection from short-read sequencing. *arXiv*:1207.3907. <https://doi.org/10.48550/arXiv.1207.3907>

Guo, Y., Betzen, B., Salcedo, A., He, F., Bowden, R. L., Fellers, J. P., Jordan, K. W., Akhunova, A., Rouse, M. N., Szabo, L. J., & Akhunov, E. (2022). Population genomics of Puccinia graminis f.sp. tritici highlights the role of admixture in the origin of virulent wheat rust races. *Nature Communications*, 13, 6287. <https://doi.org/10.1038/s41467-022-34050-w>

Kamvar, Z. N., Tabima, J. F., & Grünwald, N. J. (2014). Poppr: An R package for genetic analysis of populations with clonal, partially clonal, and/or sexual reproduction. *PeerJ*, 2, e281. <https://doi.org/10.7717/peerj.281>

Kassambara, A. (2017). Practical guide to cluster analysis in R: Unsupervised machine learning. Poland: STHDA.

Knaus, B. J., & Grunwald, N. J. (2017). vcfr: A package to manipulate and visualize variant call format data in R. *Molecular Ecology Resources*, 17, 44–53. <https://doi.org/10.1111/1755-0998.12549>

Li, H. (2013). Aligning sequence reads, clone sequences and assembly contigs with BWA-MEM. *arXiv*:1303.3997. <https://doi.org/10.48550/arXiv.1303.3997>

Martin, M. (2011). Cutadapt removes adapter sequences from high-throughput sequencing reads. *EMBnet.journal*, 17, 10–12. <https://doi.org/10.14806/ej.17.1.200>

Poplin, R., Ruano-Rubio, V., DePristo, M. A., et al. (2017). Scaling accurate genetic variant discovery to tens of thousands of samples. *bioRxiv*, 201178. <https://doi.org/10.1101/201178>

Schwessinger, B., & Rathjen, J. P. (2017). Extraction of high molecular weight DNA from fungal rust spores for long read sequencing. In S. Periyannan (Ed.), *Wheat rust diseases*. *Methods in Molecular Biology* (Vol. 1659, pp. 49–57). Humana Press, New York, NY. <https://doi.org/10.1007/978-1-4939-7249-4_5>
